# Supplementary material for: Seminoma and Embryonal Carcinoma Footprints Identified by Analysis of Integrated Genome-Wide Epigenetic and Expression Profiles of Germ Cell Cancer Cell Lines
Source: PLoS One. 2014 Jun 2;9(6):e98330. doi: 10.1371/journal.pone.0098330 (PMC4041891; doi:10.1371/journal.pone.0098330)
Supplement: File S1 — ZIP file containing DMRforPairs output for significant regions. Please start from the html files. (ZIP) [file pone.0098330.s008.zip › figures/21003.pdf]

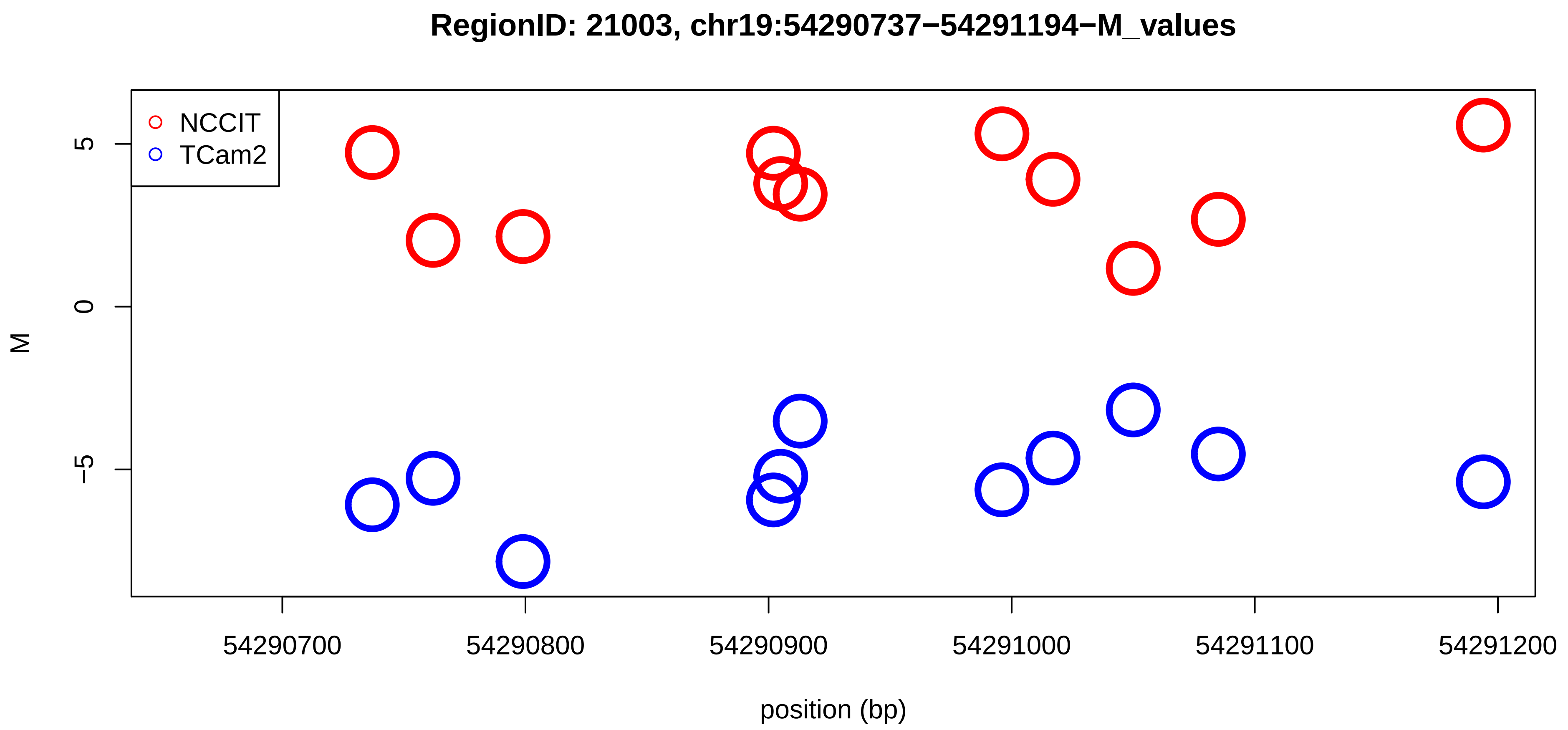

RegionID: 21003, chr19:54290737−54291194−M_values
NCCIT
TCam2
M
5
0
−5
54290700
54290800
54290900
54291000
54291100
54291200
position (bp)

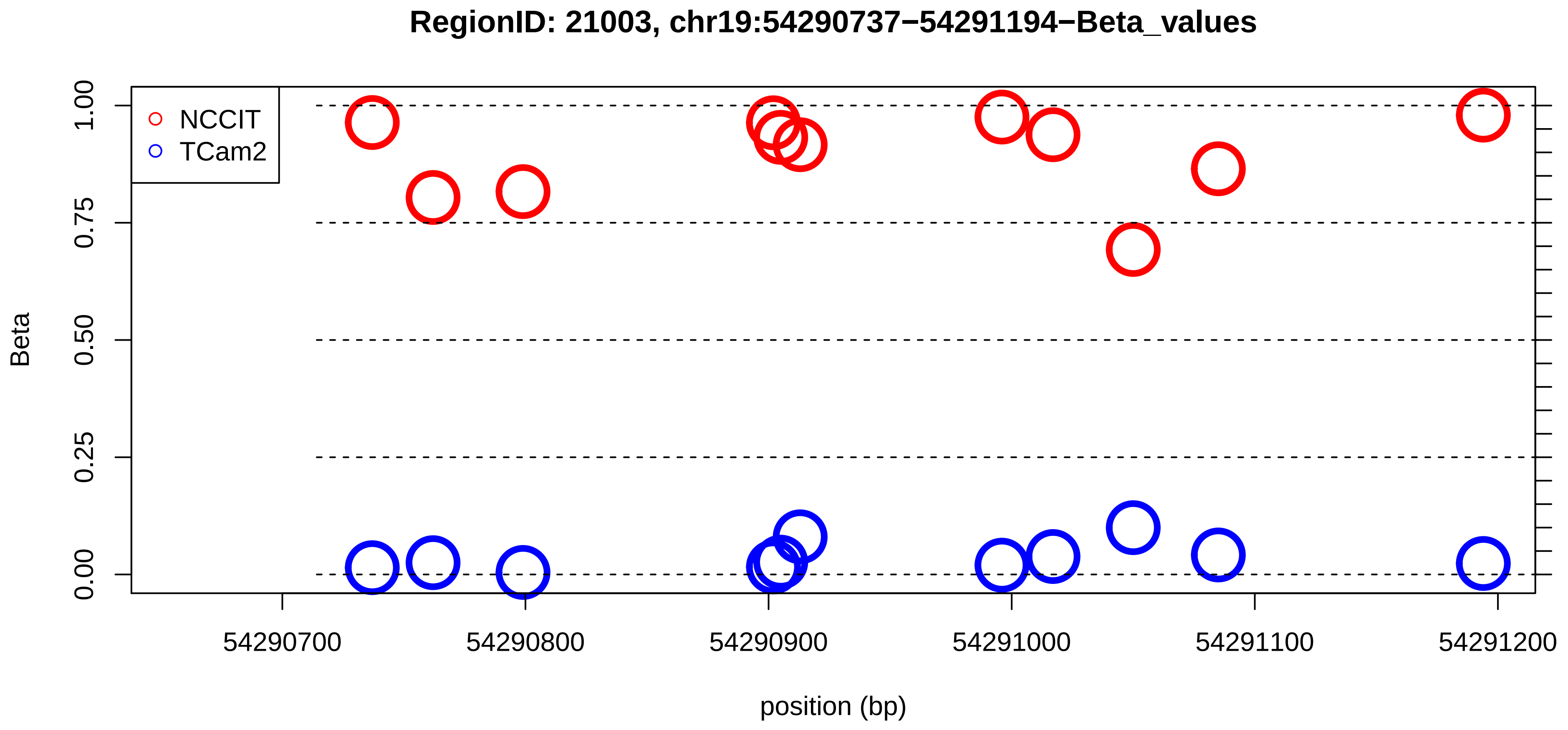

RegionID: 21003, chr19:54290737−54291194−Beta_values
NCCIT
TCam2
Beta
1.00
0.75
0.50
0.25
0.00
54290700
54290800
54290900
54291000
54291100
54291200
position (bp)

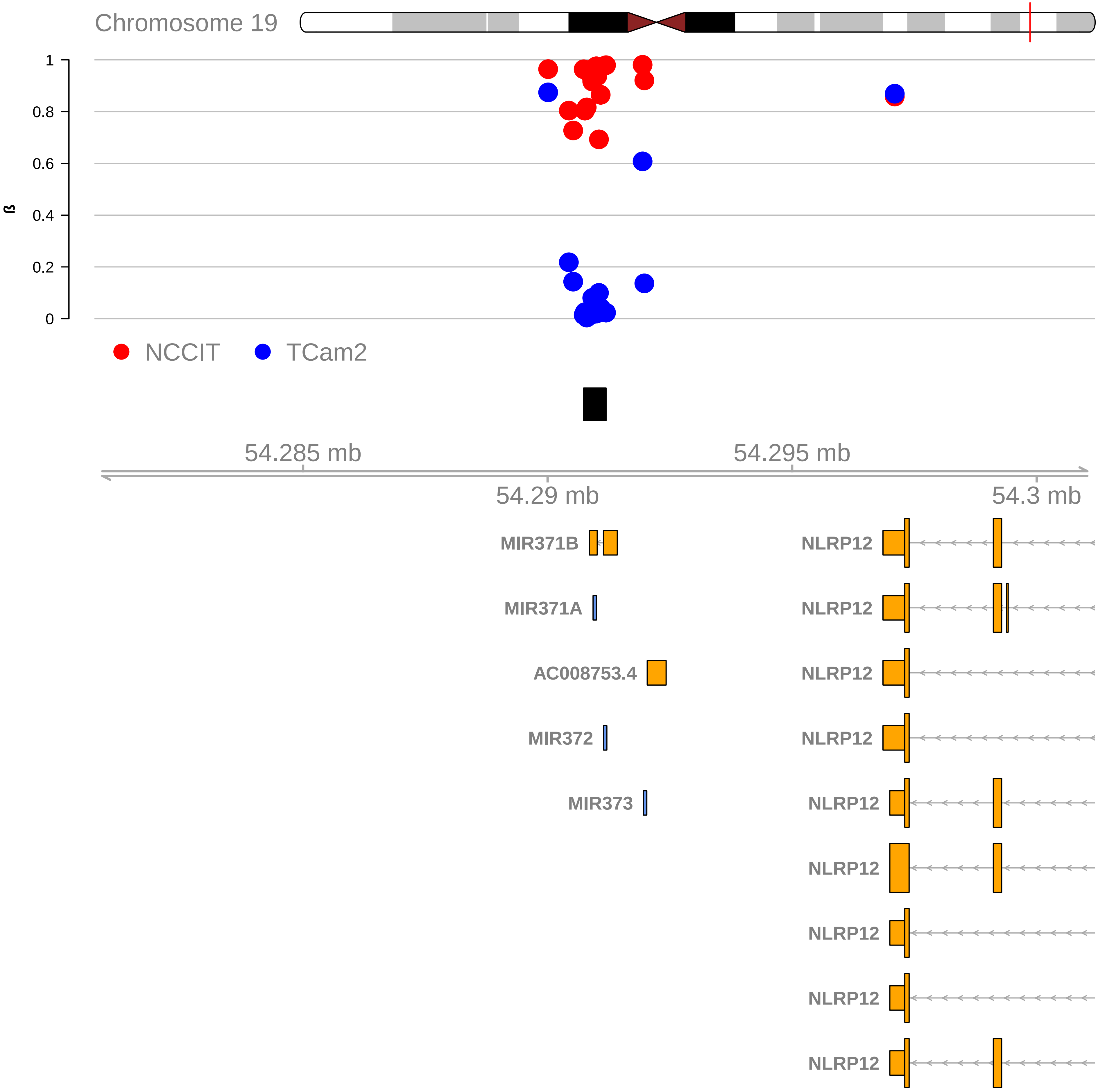
Chromosome 19
1
0.8
0.6
0.4
0.2
0
β
NCCIT
TCam2
54.285 mb
54.295 mb
54.29 mb
54.3 mb
MIR371B
MIR371A
AC008753.4
MIR372
MIR373
NLRP12
NLRP12
NLRP12
NLRP12
NLRP12
NLRP12
NLRP12
NLRP12
NLRP12
